# Synergistic Effects of Ceramic Fillers and NaOH Treatment on Bioactivity of 3D-Printed Poly(ε-caprolactone) Scaffolds for Periodontal Tissue Regeneration

**DOI:** 10.1021/acsomega.5c09061

**Published:** 2026-01-19

**Authors:** João de Freitas Gomes Neto, Bruno Pinto Moura, Tainara de Paula de Lima Lima, Felippe Migliato Marega, Leonardo Alves Pinto, Pedro L. Granja, Luiz Antonio Pessan, Eduardo Henrique Backes

**Affiliations:** † Graduate Program in Materials Science and Engineering, Federal University of São Carlos, São Carlos 13565-905, SP , Brazil; ‡ Department of Materials Engineering, UFSCar, São Paulo 13565-905, Brazil; § Biofabrication Group, i3S−Instituto de Investigação e Inovação em Saúde (i3S), Universidade do Porto, Porto 4200-135, Portugal

## Abstract

Porous scaffolds composed of poly­(ε-caprolactone) (PCL) and ceramic fillers—hydroxyapatite (HAp) and β-tricalcium phosphate (β-TCP)—were fabricated via extrusion-based additive manufacturing for bone tissue engineering applications. The scaffolds exhibited interconnected pores (∼400 μm) in a 0°–90° deposition pattern and were subjected to alkaline surface treatment with 1 M NaOH to increase surface roughness and promote partial exposure of embedded ceramic particles. Characterization included rheological analysis to assess processability, thermal evaluation via thermogravimetric analysis and differential scanning calorimetry, and surface morphology using scanning electron microscopy, energy-dispersive spectroscopy, and atomic force microscopy. Filler particle size was characterized, confirming submicron dimensions favorable for bioactive interaction; however, distribution within the polymer matrix was not directly evaluated. Mechanical testing under uniaxial compression revealed that ceramic addition increased stiffness and compressive strength. Protein adsorption assays indicated a significant increase in surface bioactivity following NaOH treatment. In vitro assays with MC3T3-E1 preosteoblastic cells confirmed good cytocompatibility, cell adhesion, and proliferation. Collectively, these findings suggest that the combination of ceramic incorporation and surface modification enhances both mechanical and biological performance, supporting the potential application of PCL-based scaffolds in bone regeneration strategies.

## Graphical abstract

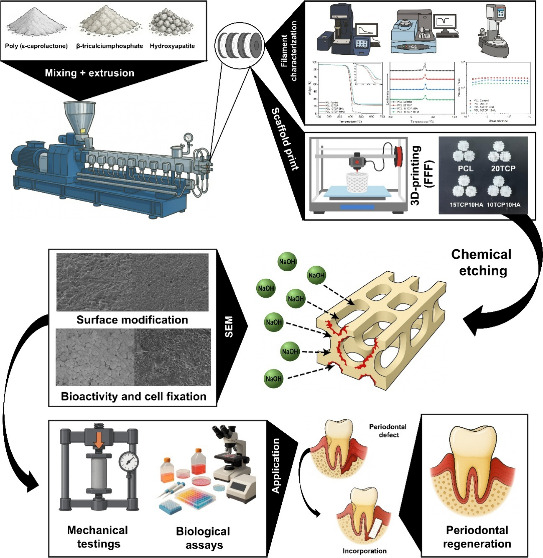

## 1 Introduction

The periodontal tissues (gingiva, periodontal ligament (PDL), alveolar bone, and cementum) form an integrated functional unit that anchors and stabilizes the teeth. Despite their morphological differences, these tissues act in a coordinated manner to maintain the structural integrity and function of the periodontium. However, due to their exposure to the oral environment, they are vulnerable to microbial action, which can lead to inflammatory conditions such as gingivitis and, in more advanced stages, periodontitis, a progressive and irreversible condition that compromises the dental supporting tissues.^1,2^ Periodontitis affects approximately 19% of the population in its severe form. It is one of the leading causes of tooth loss in adults, with a global impact estimated at over 100 million cases.^3,4^ Conventional treatments such as scaling, root planning, and surgical procedures aim to control inflammation and remove bacterial biofilms.^5^ However, these interventions are limited in their ability to regenerate the lost periodontal tissues.^6,7^ The rapid migration of epithelial cells and gingival fibroblasts to the injured area often prevents colonization by progenitor cells, compromising tissue repair and bone regeneration.^8^ As a result, functional recovery of the periodontium is incomplete, with a high incidence of relapses, clinical instability, and postoperative infections.^9^

Given the limitations of conventional treatments, tissue engineering has emerged as a promising approach for periodontal regeneration. Innovative strategies have been developed using three-dimensional (3D) scaffolds that mimic the native microenvironment of injured tissues. These scaffolds not only provide structural support but also directly influence cellular behavior, promoting the adhesion, proliferation, and differentiation of cells required for tissue regeneration.^10,11^ Among the biomaterials employed in scaffold fabrication, poly-ε-caprolactone (PCL) stands out due to its excellent biocompatibility, biodegradability, mechanical flexibility, and ease of processing.^12^ Its slow degradation rate is particularly advantageous in regeneration processes that require prolonged support, such as those involving periodontal tissues.^13^ Moreover, PCL is compatible with additive manufacturing techniques, such as 3D printing via fused filament fabrication (FFF), which enables the fabrication of scaffolds with controlled porosity (pore size, shape, and interconnectivity) essential for nutrient diffusion.^14,15^ The ability to tailor material geometry and surface topography allows the development of scaffolds with mechanical and structural properties adapted to the specific needs of each periodontal defect, contributing to more effective and personalized regeneration.^16,17^ However, when used alone, PCL lacks the intrinsic osteoinductive capacity required to initiate bone formation and to overcome this limitation. To overcome this limitation, several research teams have focused on improving bone defect repair by incorporating ceramic fillers into PCL scaffolds, particularly through the combination of PCL with osteoconductive bioceramics such as hydroxyapatite (HAp) and β-tricalcium phosphate (β-TCP), aiming to enhance both the mechanical properties and the biological performance of the material.^18,19^

HAp is highly valued due to its chemical composition, which closely resembles the mineral phase of natural bone and teeth, thereby favoring biological integration. This calcium phosphate-based ceramic stands out for its biocompatibility, bioactivity, and ability to support the attachment and growth of bone cells.^20^ β-TCP exhibits remarkable osteoconductive properties and osteoinductive potential. This material degrades gradually within the body, releasing calcium (Ca^2+^) and sulfate (SO_4_^2–^) ions, which are essential for bone formation. This process supports both the repair of damaged areas and the formation of new bone tissue. However, its degradation rate may not always align with the pace of new bone formation, which could result in premature resorption and compromise the regenerative outcome.^21^

Alkaline surface treatments, particularly with sodium hydroxide (NaOH), have been widely used to improve the biointerfacial properties of PCL-based scaffolds. Yeo et al.^22^ reported that treating PCL-20TCP scaffolds with 3 M NaOH for 48–96 h significantly increased surface roughness, exposed TCP particles, and enhanced interfacial mechanical interlock and early bone formation in vivo. Similar NaOH etching strategies have been employed to improve the hydrophilicity and cell attachment of 3D-printed PCL scaffolds, confirming that alkaline hydrolysis is an effective and versatile route to tune the surface of PCL-based constructs for bone repair. Building on this, Lam et al.^23^ demonstrated that alkaline hydrolysis of PCL and PCL−β-TCP scaffolds in 5 M NaOH at 37 °C led to progressive surface erosion, increased porosity, and a marked decrease in mechanical properties, with complete degradation of PCL–20TCP constructs occurring within 54 h. This was attributed to the preferential leaching of β-TCP particles, which facilitated micropore formation and accelerated hydrolytic breakdown. Complementarily, Wang et al.^24^ applied NaOH treatment to stretched PCL films and reported enhanced surface roughness and increased wettability, which significantly improved cell adhesion and guided alignment of human mesenchymal stem cells. These findings reinforce that NaOH modification is not only effective in tuning degradation kinetics but also in promoting biofunctional surface cues for cellular response and tissue integration.

Some studies have shown promising results regarding the combination of the polymer with the fillers. Wu et al.^25^ investigated how PCL/β-TCP scaffolds with different compositions behave differently at each stage of degradation during bone regeneration. In their study, PCL and β-TCP were mixed in five ratios (70:30, 60:40, 50:50, 40:60, and 30:70) and processed via melt extrusion. These materials were 3D-printed into porous scaffolds in three shapes: discs for in vitro and subcutaneous tests, cylinders for femoral implantation, and cubes for material characterization and mechanical testing. The results showed that the PCL/β-TCP ratio directly affects the scaffolds’ physicochemical, mechanical, and biological properties. Scaffolds with a higher β-TCP content (e.g., 30:70) exhibited greater stiffness, enhanced osteogenic and angiogenic activity in vitro, and greater Ca^2+^ ion release. However, their rapid in vivo degradation disrupted the immune response, leading to M1-type macrophage polarization and impairing bone regeneration. In contrast, intermediate compositions, such as 50:50, promoted better cell adhesion, expression of osteogenic markers, and a more favorable immune response, characterized by M2 macrophage polarization. Therefore, the PCL/β-TCP ratio must be carefully adjusted according to the regenerative phase, taking into account the impact of degradation on the immune response and bone formation. Bruyas et al.^26^ analyzed the influence of porosity and β-TCP filler content in 3D-printed PCL scaffolds for potential bone-substitute applications. In the study, PCL/β-TCP mixtures were prepared in four different ratios (100:0, 80:20, 60:40, and 40:60) and dissolved in dimethylformamide at 80 °C. After stirring, the solution was precipitated in water to remove the solvent, dried at room temperature for 24 h, and manually cut into 5 mm pellets. The PCL/β-TCP pellets were then melted at 90 °C and extruded into filaments for 3D printing. Five porosity levels were also evaluated, adjusted by filament spacing (0.4, 0.5, 0.71, 1.25, and 2.5 mm). The results showed that surface roughness increased and the contact angle decreased as the β-TCP content rose. The degradation rate also intensified with higher ceramic content. Regarding biological behavior, β-TCP promoted both proliferation and osteogenic differentiation of C3H10 cells. Subsequently, the influence of composition and porosity on the mechanical properties of the 3D-printed scaffolds was systematically assessed. It was observed that increasing β-TCP content and decreasing porosity resulted in higher stiffness, as indicated by an increase in the apparent Young’s modulus. In the work of Rezania et al.,^27^ scaffolds composed of PCL and four different proportions of HAp (5, 10, 15, and 20 wt %) were developed. The study aimed to evaluate the effect of HAp addition on the mechanical properties, bioactivity, and in vitro biological behavior of scaffolds produced using a low-cost commercial 3D printer. The results showed that incorporating HAp significantly increased the stiffness and compressive strength of the filaments and scaffolds, with the 20% HAp formulation exhibiting a 50% increase in Young’s modulus compared to pure PCL. Furthermore, biological tests demonstrated good cytocompatibility in all samples, with the presence of HAp enhancing cell adhesion, alkaline phosphatase (ALP) activity, and calcium phosphate deposition. Apatite formation was also observed on the surface of scaffolds immersed in SBF, especially in formulations with higher HAp content. Backes et al.^12^ also investigated the influence of incorporating three different concentrations of HAp (5, 10, and 25 wt %) into the PCL matrix, aiming to develop composites with suitable physicochemical, mechanical, rheological, and biological properties for the fabrication of scaffolds via 3D printing, with potential application in bone tissue regeneration. The results showed that the addition of 5 and 10% HAp to PCL maintained good processability, improved elastic modulus, and supported osteoblast proliferation. The scaffolds exhibited a uniform structure, with properties comparable to cancellous bone, indicating their promise for bone regeneration applications.

In parallel with advances in extrusion-based additive manufacturing, recent studies have highlighted the emergence of so-called hyperelastic biodegradable composites as a new class of biomaterials for bone and craniofacial regeneration. These systems, typically based on elastomeric or semicrystalline polymer matrices combined with high fractions of bioactive ceramics, are designed to exhibit nonlinear elastic deformation, high recoverability, and shape conformity while maintaining osteoconductive potential.^28^ Previous studies^29,30^ demonstrated that ceramic-rich, highly deformable 3D-printed constructs could reconcile surgical handling, defect filling, and biological performance, giving rise to the concept of “hyperelastic bone”. Subsequent studies have shown that, in such systems, mechanical behavior is strongly influenced by hydration, interfacial interactions, and early stage degradation, emphasizing the importance of understanding how scaffold mechanics evolve under physiologically relevant conditions. Although the present study does not aim to develop a fully hyperelastic construct, these findings are highly relevant, as they underscore the need to balance printability, mechanical integrity, and bioactivity in biodegradable polymer–ceramic composites intended for periodontal and bone regeneration. Within this framework, evaluating the mechanical response under controlled dry conditions provides a necessary baseline to elucidate the role of ceramic fillers and surface modification, while future studies under hydrated environments can further expand the understanding of time-dependent mechanical behavior.

In addition, clinical trials have already been conducted using these materials, such as the study by Wong et al.,^31^ which reported the development and clinical application of a personalized synthetic bone. The device, named Osteopore wedge, was produced by 3D printing from a biocomposite of PCL and β-TCP in an 80:20 ratio. The implant was custom-made for each patient and inserted into the osteotomy gap using a press-fit technique, eliminating the need for autologous or allogenic bone grafts. Radiographic and tomographic results demonstrated progressive integration and neo-cortex formation between 6 and 12 months, indicating bone remodeling and regeneration. Similarly, Laubach et al.^32^ conducted clinical studies using biodegradable medical-grade PCL-TCP (80:20) *scaffolds* produced by 3D printing and designed for the specific anatomy of each bone defect. The porous structure, featuring a triangular pattern of 2–3 mm, provided a favorable microenvironment for cell migration, vascularization, and progressive bone formation. Clinical outcomes showed good tissue integration, absence of inflammation, and continuous bone formation within and around the *scaffold*. In one case, complete bone fusion and full functional recovery were observed after 23 months. Those study highlights that the combination of personalized design, controlled biodegradability, and the use of autologous grafts significantly enhances the regeneration of large and irregular bone defects.

As observed, the use of PCL composites with HAp or β-TCP has been widely reported for applications in bone tissue engineering. However, studies exploring the combination of these two ceramic fillers within the same polymeric matrix remain scarce, particularly with a focus on periodontal regeneration. In this context, the present work aims to investigate different proportions of HAp and β-TCP incorporated into PCL to develop scaffolds with optimized physicochemical, mechanical, and biological properties. The proposed approach integrates the precision of 3D printing with the bioactivity of the selected materials to meet the structural and functional requirements of periodontal tissue regeneration.

## 2 Materials and Methods

### 2.1 Materials

Poly­(ε-caprolactone) (PCL) with a molar mass of 50,000 g mol^–1^ was supplied by Perstorp (CAPA 6500, Sweden) and used without further modifications. According to the manufacturer, it presents a melt flow index of 7.9 g/10 min (160 °C, 2.16 kg, ASTM D-1238), a melting temperature of approximately 60 °C, and a glass transition temperature (*T*_g_) of −60 °C. Hydroxyapatite (HAp) was provided by Sigma-Aldrich (ref 21223; USA), with a purity greater than 90% and particle size distribution (*d*_50_) of 3.7 μm. β-tricalcium phosphate (β-TCP) was purchased from Fluidinova (NanoXIM-TCP200; Portugal), with a chemical formula of Ca_3_(PO_4_)_2_ and a purity greater than 90%. The particle size distribution (*d*_50_) was 4.0 μm. Prior to use, the TCP was sintered at 1000 °C for 1 h in a laboratory muffle furnace (Jung, model LF0916; Brazil). The SBF solution was prepared by dissolving analytical grade reagents (NaCl, NaHCO_3_, KCl, K_2_HPO_4_·3H_2_O, MgCl_2_·6H_2_O, CaCl_2_, Na_2_SO_4_, and TRIS) in distilled and deionized water under continuous stirring and temperature control at 36.5 ± 1.5 °C. The order of reagent addition was strictly followed to prevent the spontaneous precipitation of calcium phosphate. The pH adjustment of the solution was carried out by carefully adding TRIS (tris­(hidroximetil)­aminometano) and 1.0 M HCl, maintaining a final pH of 7.40 ± 0.01 at a constant temperature of 36.5 ± 0.2 °C.

### 2.2 Methods

#### 2.2.1 Particle Size Distribution

A laser diffraction particle size analyzer (Cilas, 1190L) was employed to evaluate both the size distribution and mean diameter of the biofiller particles. For analysis, the material was dispersed in water containing 1 wt % Darvan as dispersant, yielding a suspension with 10 wt % solids. This suspension was then treated in a probe-type ultrasonic device (VCX 500, Sonics) for 15 min to promote complete breakup of particle agglomerates.

#### 2.2.2 Filament Fabrication

Initially, PCL pellets underwent cryogenic milling to reduce particle size, facilitate subsequent extrusion, and improve the efficiency of mixing with ceramic fillers. Milling was carried out using a cryogenic grinder operated with liquid nitrogen, aiming to minimize thermal degradation of the PCL. The resulting powder was collected, dried, and stored under controlled temperature and humidity conditions until the extrusion process.

A corotating twin-screw extruder (MT19TC; B&P Process Equipment and Systems, USA), with a length-to-diameter (L/D) ratio of 25 and screw diameter of 19 mm, was employed for filament fabrication. The materials were extruded at a screw speed of 20 rpm, with a temperature profile ranging from 85 °C up to 135 °C, from the feed zone to the die. Filaments with a diameter of 1.7 ± 0.1 mm were obtained by adjusting the winding speed and material flow rate.^12,33^

Four different filament compositions were prepared: neat PCL (PCL CONTROL); PCL with 20 wt % of TCP (PCL20TCP); PCL with 15 wt % TCP and 5 wt % HAp (PCL15TCP5HA); and PCL with 10 wt % TCP and 10 wt % HAp (PCL10TCP10HA) (Table 1). The selection of these compositions was based on previous studies,^34^ which demonstrated promising results regarding processability and biological performance of composites containing up to 20 wt % TCP. Higher concentrations often led to flow instabilities during extrusion and 3D printing. Furthermore, one of the main hypotheses of this study is to evaluate the effect of combining two distinct ceramic fillers — TCP and HAp — on the rheological, thermal stability, bioactivity, and printing properties of the resulting scaffolds.

**Table 1:** Compositions of PCL-Based Composites with Ceramic Fillers (TCP and HAp)

sample	PCL (wt %)	β-TCP (wt %)	HAp (wt %)
PCL control	100	0	0
PCL20TCP	80	20	0
PCL15TCP5HA	80	15	5
PCL10TCP10HA	80	10	10

#### 2.2.3 Scaffolds 3D Printing

Three-dimensional porous structures based on PCL filaments and PCL-based biocomposites with different HAp/TCP contents were fabricated using a custom 3D printer (Sethi S3; Brazil). The 3D scaffolds to be printed were designed through computer-aided design (CAD) software, specifically SolidWorks (Dassault Systèmes S.A, France).

The samples for analysis were fabricated in a cylindrical shape, with dimensions of 8 mm in diameter and 3 mm in height. The scaffolds were fabricated with interfilament pores of 400 μm and an alternating 0°–90° deposition pattern. These geometrical and structural characteristics followed widely adopted parameters in the literature, which demonstrated that the 0–90° deposition pattern not only enables the fabrication of regular and interconnected porosity, a key requirement for nutrient diffusion and cell infiltration, but also facilitates the standardization of in vitro assays and comparability with previous studies.^35^ These studies have shown that the orthogonal layer arrangement promotes cell adhesion and proliferation, while also providing a simplified structural mimicry of the trabecular bone architecture. Therefore, the adoption of this pattern enhances the robustness of the bioactivity and cytocompatibility assays to be performed.

The 3D models of the scaffolds were sliced using the free PrusaSlicer (Prusa Research, Czech Republic) software, and printing was controlled via the open-source Repetier-Host (Hot-World GmbH & Co. KG.) platform, compatible with the Sethi3D S3 printer used. Several printing parameters, including temperature, sample quantity, and cooling conditions (Table 2), were optimized to ensure the fabrication of structures that closely match the original CAD design.

**Table 2:** 3D Printing Parameters and Configurations Applied during the Manufacturing Process

parameter	configuration
printing temperature	90 °C
printing speed	30 mm.s^–1^
travel speed	150 mm.s^–1^
layer height	0.3 mm (300 μm)
layer width	0.4 mm (400 μm)
infill	42%
raster angle	0°/90°
perimeters (shell)	without shell
print bed temperature	25 °C (room temperature)

#### 2.2.4 Rheological Characterization

The rheological behavior of the PCL filaments and PCL-based biocomposites was evaluated at low shear rates and within the temperature ranges corresponding to their respective 3D printing conditions. Steady-state shear viscosity measurements were performed using a stress-controlled rheometer (AR-G2, TA Instruments, EUA) equipped with a parallel plate geometry (25 mm diameter, 1 mm gap) under an inert nitrogen atmosphere.

The viscoelastic characterization under oscillatory shear was carried out under the same experimental conditions used for low shear rate analysis. In this step, strain amplitudes ranging from 0.1 to 10% were applied, within the linear viscoelastic region (LVR) of each material. The storage modulus (G′) and loss modulus (G″) were determined as a function of angular frequency, varying from 0.1 to 500 rad/s. This approach aimed to assess the processability of the materials via fused filament fabrication (FFF), verifying whether the obtained rheological properties were suitable for 3D printing. Additionally, we investigated whether ceramic filler-induced degradation phenomena occurred during biocomposite processing, which could lead to a reduction in the viscosity of the extruded material.

#### 2.2.5 Thermal Characterization

The thermal stability of PCL, the bioceramic fillers, as well as the composite filaments was evaluated using thermogravimetric analysis (TGA). The experiments were conducted on a TGA Q50 system (TA Instruments, EUA), operating at a heating rate of 20 °C.min^–1^, from room temperature to 800 °C, under a nitrogen atmosphere.

The objective of this analysis is to investigate the effect of ceramic filler incorporation on the thermal behavior of the composite filaments by determining parameters such as the onset degradation temperature (*T*_onset_) and the temperature at maximum degradation rate (*T*_max_), the latter obtained from the derivative of the mass loss curve as a function of temperature.

The differential scanning calorimetry (DSC) protocol for PCL and ceramic-filled biocomposites aims to evaluate the thermal properties of these materials, including melting temperature (*T*_m_) and degree of crystallinity, to investigate possible interactions between the polymer matrix and the ceramic fillers. Filament scaffolds of approximately 5–10 mg, were placed in sealed aluminum pans. DSC measurements were performed under a nitrogen flow of 20–50 mL.min^–1^ over a temperature range of −70 to 150 °C, using a heating rate of 10 °C.min^–1^.

The test included thermal cycling, which consisted of an initial heating run to eliminate the previous thermal history, followed by controlled cooling and a second heating scan for detailed analysis. From the acquired data, the *T*_m_ was determined, and the degree of crystallinity (*X*_c_) was calculated according to eq 1:

$$X_{c}(\%)=\frac{\Delta H_{m}}{\Delta H_{m}^{0}\times w\mathrm{PCL}}$$ 1

Where Δ*H*_m_ corresponds to the measured melting enthalpy, Δ*H*_m_^0^ to the enthalpy of fusion for 100% crystalline PCL (139.5 J.g^–1^), and *w*PCL to the effective weight fraction of PCL in the composite, accounting for the ceramic filler content. The results were compared with those of neat PCL to assess the influence of ceramic fillers on the material’s thermal properties, such as shifts in *T*_m_ or variations in crystallinity, which may indicate specific interactions between the polymer and the fillers.

#### 2.2.6 Surface Treatment

Surface treatment of the scaffolds was performed through a controlled chemical etching process using a 1 M NaOH solution, which is aimed at modifying the material’s surface and exposing the embedded ceramic fillers. This exposure is described as enhancing the bioactivity of the scaffold. This methodology was chosen due to its simplicity, low cost, and high reproducibility, making it suitable for both laboratory studies and potential large-scale applications.

For the treatment, the samples were fully submerged in a container filled with the NaOH solution, ensuring complete immersion, and maintained under these conditions for 8 h at room temperature with mild agitation. Other protocols with different times and concentrations were tested, and this one was selected because it provided suitable surface modification properties without severely compromising the mechanical stability. Samples subjected to surface treatment will be designated with the suffix _T after their name. During the process, the solution was kept homogeneous. At the end of the established time, the samples were carefully removed and rinsed repeatedly with distilled or deionized water until the rinsing water reached neutral pH, ensuring the complete removal of residual NaOH. After rinsing, the scaffolds were dried in an oven at a controlled temperature (∼40–45 °C) until they reached a constant weight. The chemical treatment was designed to modify the scaffold surface topography, promoting exposure of ceramic particles and enhancing calcium phosphate formation upon contact with body fluids.

#### 2.2.7 Atomic Force Microscopy (AFM)

The topographical characterization of scaffold surfaces was carried out by atomic force microscopy (AFM) in Tapping Mode in Air (Soft Tapping) using an AFM system (Model XYZ, Bruker, Billerica, MA, USA). Measurements employed the Tap150A probe (P/N MPP12120–10, Bruker), which is suitable for the selected application. Samples were affixed to AFM stubs using Araldite adhesive to ensure firm positioning and stability during scanning. Between analyses, samples were stored in a desiccator to prevent moisture absorption and potential interference with imaging. The image acquisition parameters were defined as follows: scan size of 5.0 μm, scan rate of 0.5 Hz, 256 scan lines, and an amplitude set point of approximately 150 mV.

#### 2.2.8 Bioactivity via Immersion in Simulated Body Fluid

The bioactivity of the fabricated 3D structures was assessed in vitro by immersion in SBF, in accordance with ISO 23317. Once prepared, the SBF was stored in suitable plastic containers under refrigerated conditions (5–10 °C) and used within 30 days.

For the bioactivity assay, the scaffolds were immersed in SBF, maintained in an incubator at 37 °C under agitation for a predetermined period (7 days). The SBF volume-to-sample surface area ratio was kept at a minimum of 10 mL.cm^–2^ to ensure sufficient ionic saturation. After immersion, the scaffolds were carefully removed, rinsed with distilled water, and air-dried at room temperature. The scaffold surfaces were then analyzed by scanning electron microscopy (SEM) to investigate the formation of calcium phosphate layers and to characterize the surface chemical composition.

#### 2.2.9 Contact Angle

Contact angle measurements were performed on hot-compressed sample plates using an optical tensiometer (Biolin Scientific, model Attension Theta Flex TF3000-Plus, EUA). For this analysis, the samples were placed on a flat surface, and 8 μL of distilled water was deposited on either the flat sample or the scaffold surface. After a 10-s waiting period, the measurement was taken, and the results were processed using the OneAttension analysis software. The contact angle values (θ) were calculated as the average of six independent measurements for each sample. This analysis aims to assess the wettability, cell, and protein adhesion potential of the scaffolds composed of neat PCL and PCL-based biocomposites.

#### 2.2.10 Protein Adhesion

The amount of bovine serum albumin (BSA) adsorbed on the scaffolds was quantified by the Pierc BCA Protein Assay Kit (Thermo Fisher Scientific, Waltham, MA, USA). The samples were placed in a 48-well plate. Then, 0.4 mL of a 2 mg·mL^–1^ BSA solution (Sigma-Aldrich, St. Louis, MO, USA) in phosphate-buffered saline (PBS) was added, and the plate was incubated at 37 °C for 24 h. The scaffolds were then transferred to a new plate, washed twice with 0.4 mL of PBS, and 0.4 mL of RIPA buffer (Sigma-Aldrich, St. Louis, MO, USA) was used for protein solubilization (10 min in contact and homogenization with pipet). In a 96-well plate, 200 μL of the working reagent was added along with 25 μL of the samples (RIPA + adsorbed proteins) or 25 μL of the prepared calibration standards containing different concentrations of BSA in RIPA. The plate was incubated at 37 °C for 30 min, cooled to room temperature, and absorbance was measured at 562 nm using a spectrophotometer (SpectraMax i3, Molecular Devices, San Jose, CA, USA). The concentration of protein was determined through a calibration curve using BSA standards.

#### 2.2.11 Microstructural Characterization

The morphological characterization of the fabricated 3D structures was performed by SEM. This analysis included the evaluation of pore size and distribution, layer thickness and geometry, overall porosity, and the distribution of the incorporated ceramic fillers, which were further investigated through energy-dispersive X-ray spectroscopy (EDS). Considering that the mechanical properties of biocomposites are strongly influenced by the morphology and dimensions of the filler particles, it is essential to ensure their homogeneous dispersion within the polymer matrix. For sample preparation, scaffolds were mounted on aluminum stubs using carbon conductive tape and coated with a thin gold layer to enhance electrical conductivity. SEM analysis was conducted using a Mira microscope (Tescan, EUA) operated at an acceleration voltage of 10 kV.

#### 2.2.12 Mechanical Characterization

The mechanical properties, including compressive strength and elastic modulus of the porous PCL structures and its biocomposites, were evaluated through uniaxial compression tests, following the ASTM D695–15 standard. The tests were performed using a universal testing machine (Instron, model 5569, EUA) equipped with a 500 N load cell, operating at a crosshead speed of 1.3 mm min^–1^, and applying a preload of 15 N.

#### 2.2.13 Biological Assay

Osteoblast proliferation on scaffolds was assessed using murine preosteoblastic MC3T3-E1 cells. Cells were cultured in a medium consisting of 89% (v/v) α-MEM (Gibco, Brazil) supplemented with 10 (wt %) fetal bovine serum (FBS; Vitrocell, Brazil) and 1 (wt %) antibiotic–antimycotic solution (Vitrocell, Brazil), maintained at 37 °C in a humidified incubator (Series II 3110, Thermo Fisher Scientific) with 5% CO_2_.

Prior to biological assays, the scaffolds were sterilized by immersion in 70% ethanol, followed by exposure to UV light on both sides for 15 min each. After sterilization, the samples were rinsed with phosphate-buffered saline (PBS) and preincubated with 500 μL of culture medium for 24 h at 37 °C. Following preincubation, the scaffolds were transferred to a new 48-well culture plate, and 500 μL of a cell suspension containing 50,000 osteoblastic cells per well was added. The culture medium was replaced every 2–3 days.

For the cell proliferation assay, a working solution was prepared by diluting resazurin solution (#R7017, Millipore-Sigma) in culture medium at a 1:9 ratio. After 1 and 7 days of cell culture, the scaffolds were transferred to a new plate, the medium was removed, and 500 μL of the working solution was added to each well. The plates were then incubated at 37 °C for 4 h in the dark. Subsequently, 100 μL of each sample solution was transferred to a transparent 96-well plate, and absorbance was measured at 560 and 590 nm using a microplate reader (SpectraMax M5). Three samples per composition were analyzed, including negative controls (resazurin solution without cells) and a positive control (autoclaved resazurin solution) to estimate cell viability.

Additionally, after 1 day of cell culture, cell adhesion and proliferation on the scaffolds were evaluated by SEM. For that purpose, the scaffolds were transferred to a new plate, washed with PBS, and fixed in 1 mL of a 2.5% glutaraldehyde solution for 30 min. Samples were then washed again with PBS and dehydrated through a graded ethanol series (50, 70, 90, and 100%). Following dehydration, the samples were dried, sputter-coated with a thin gold layer, and analyzed by SEM using a TESCAN MIRA FEG microscope operated at an acceleration voltage of 5 kV.

#### 2.2.14 Statistical Analysis

Data are presented as mean ± standard deviation and analyzed using one-way analysis of variance (ANOVA) followed by Tukey’s post hoc test, implemented in Python with the Pingouin and Pandas libraries. Statistical significance was set at *p* < 0.05. In the figures, *p* < 0.05, *p* < 0.01, and *p* < 0.005 are denoted by *, **, and ***, respectively.

## 3 Results and Discussion

### 3.1 Particle Size Distribution

The particle size distribution analysis provided valuable insight into the physical characteristics of the β-TCP and HAp powders prior to incorporation into the PCL matrix. As shown in Figure 1, the β-TCP particles (a) displayed a relatively broad size distribution, with a peak centered between approximately 1 and 10 μm, indicating a predominantly microscale structure. In contrast, the HAp particles (b) presented a narrower distribution, with most particles falling within the submicron to low micrometer range. These differences in size distributions could influence not only the particle dispersion within the polymeric matrix but also their interfacial interactions with PCL during processing. Additionally, smaller particles, such as HAp, may provide a greater surface area for protein adsorption or hydrolysis reactions when exposed on the scaffold surface. However, due to the lack of direct morphological imaging (e.g., SEM or TEM of the powders), further studies are required to confirm particle shape and aspect ratio, which are also known to affect composite properties.^36^

**Figure 1:**
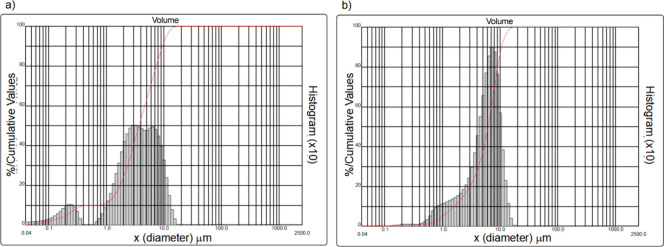
Particle size distribution curves of the ceramic powders used in this study. (a) β-tricalcium phosphate (β-TCP) and (b) hydroxyapatite (HAp) powders.

### 3.2 Rheological Performance

The rheological analysis of the PCL-based composites revealed distinct trends in viscosity as a function of shear rate, highlighting the influence of ceramic content and type on the material’s flow properties (Figure 2). All samples exhibited relatively constant viscosities across the tested shear rate range (∼10^–2^ to 10^1^ s^–1^), suggesting a predominantly Newtonian behavior under these conditions. This finding is consistent with previous studies that observed similar responses in melt-processed PCL/ceramic composites at low to moderate ceramic loadings, where the onset of shear thinning is suppressed by the dominance of viscous flow in the polymer matrix.^33,37^

**Figure 2:**
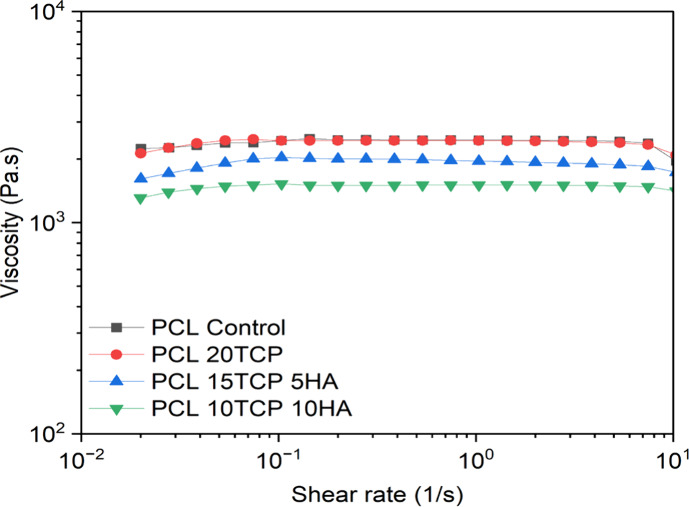
Viscosity versus shear rate for poly­(ε-caprolactone)-based composites.

Notably, the control sample (PCL Control) and the PCL20TCP formulation presented the highest viscosities, with minimal deviation across the shear rate spectrum. The increased viscosity observed in the PCL20TCP composite is likely attributable to the high ceramic loading, which restricts polymer chain mobility and increases resistance to flow.

In contrast, the formulations containing both β-TCP and HAp exhibited lower viscosities compared to PCL20TCP, with PCL10TCP10HA showing the lowest values across all shear rates. This suggests a possible synergistic interaction in the competitive interaction between the two ceramic fillers, which may influence dispersion, interfacial interactions, and, consequently, the rheological response. According to Beatrice et al.,^33^ excessive ceramic content (e.g., ≥25 wt %) can lead to agglomeration, which increases viscosity, but moderate amounts (≤10 wt %) tend to be well dispersed and minimally disruptive to flow behavior. Therefore, the reduced viscosity in the dual-ceramic systems may reflect reduced particle-filler and filler–filler interactions.

Despite the variations in viscosity, all compositions maintained viscosities within the range suitable for FFF, which typically requires values between 10^2^ and 10^4^ Pa·s to ensure smooth extrusion and shape fidelity.^38^ The nearly flat viscosity curves suggest stable flow behavior under typical FFF shear conditions, and the absence of pronounced shear-thinning behavior is beneficial for dimensional control during printing. This is supported by prior work showing that well-dispersed PCL/TCP scaffolds could be printed with high fidelity using conventional extrusion-based systems.^39^

The frequency sweep tests shown in Figure 3 provide insight into the viscoelastic behavior of the PCL-based composites under oscillatory shear. Across all samples, the complex viscosity (η*) exhibited relatively flat profiles over the angular frequency range (Figure 3a), reaffirming the near-Newtonian behavior observed in steady-state flow (Figure 2). The formulation containing 20 wt % β-TCP presented the highest η* values, especially in the low-frequency regime, suggesting enhanced melt resistance and network formation, likely due to increased particle-filler and filler–filler interactions and particle clustering. This behavior is consistent with the findings of,^33^ who reported that high TCP loading (≥25 wt %) in PCL significantly elevates the melt viscosity and impairs flowability due to the formation of agglomerates.

**Figure 3:**
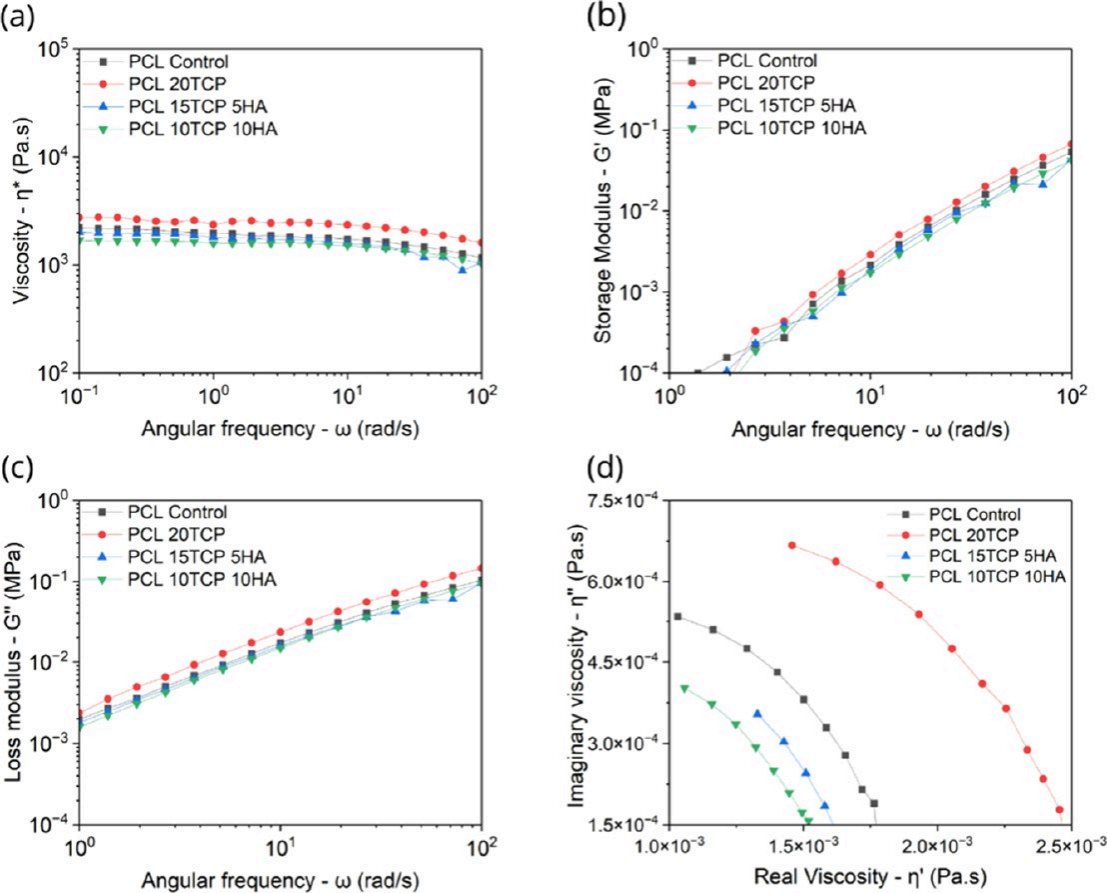
Dynamic rheological properties of poly­(ε-caprolactone)-based composites: (a) complex viscosity (η*), (b) storage modulus (G′), (c) loss modulus (G″), and (d) Cole–Cole plot.

The storage modulus (G′) and loss modulus (G″), presented in Figure 2b,c, respectively, increased monotonically with frequency for all samples. At low frequencies, G″ exceeded G′, indicating that the systems behave predominantly as viscous fluids. However, no crossover between the two moduli was observed within the tested frequency range, which is typical for thermoplastic melts with low elasticity and no yield stress at processing temperatures. The PCL20TCP composite consistently showed higher G′ across the samples, indicating a more elastic and dissipative behavior. Similar results were reported by Huang and Bártolo,^37^ who demonstrated that increasing ceramic content in polymer blends enhanced both energy storage and dissipation capabilities by restricting molecular mobility and increasing structural stiffness.

Interestingly, the dual-filler systems (PCL15TCP5HA and PCL10TCP10HA) presented intermediate values of G′ and G″ between the control and PCL20TCP. This suggests that the inclusion of HAp may modulate the viscoelastic response due to its distinct physicochemical characteristics. Although HAp typically has high purity (>90%), its crystallinity is lower, and its trace metal content, especially iron and alkaline metals, is reduced (<400 ppm), which may influence its reactivity during thermal processing compared to β-TCP. In addition, HAp has a higher specific surface area, offering more interfacial contact with the PCL matrix, which could enhance dispersion but also potentially catalyze degradation reactions. These opposing effects (greater dispersion versus increased susceptibility to degradation) may explain the intermediate rheological behavior observed in the dual-filler systems. Backes et al.^39^ also noted that HAp incorporation at low to moderate concentrations improved homogeneity without markedly increasing the elastic resistance of the melt. This balanced viscoelastic profile reinforces the potential of these hybrid formulations for extrusion-based additive manufacturing, where both flowability and structural integrity are essential.

The Cole–Cole plot (Figure 3d), representing imaginary versus real viscosity (η″ vs. η′), offers further insight into the melt relaxation dynamics of the composites. All samples showed a typical semicircular arc, characteristic of materials with a single relaxation time or a relatively narrow relaxation spectrum. The PCL control and PCL20TCP composites occupied the upper right region of the plot, indicating higher viscous and elastic contributions. In contrast, the PCL10TCP10HA formulation was located in the lower left quadrant, suggesting faster relaxation and reduced resistance to flow. Taken together, the dynamic rheological results confirm that filler composition and distribution significantly impact the viscoelastic behavior of PCL-based composites. While high β-TCP loading increases resistance to deformation and enhances elastic recovery, it may hinder printability by raising viscosity and slowing relaxation. On the other hand, combining TCP with HAp at moderate ratios appears to preserve favorable flow characteristics while improving structural response, an advantageous compromise for 3D printing applications in periodontal tissue regeneration.

### 3.3 Thermal Behavior

Thermal stability is a key requirement for materials used in 3D printing, as thermal degradation during processing can impair material flow and, consequently, compromise the quality of the printed samples. To assess this parameter, thermogravimetric analysis (TGA) was performed on HAp and β-TCP ceramic powders, the pure PCL filament, and the PCL-based composites.

Figure S1 shows the TGA curves of the ceramic powders, indicating that β-TCP maintained its mass virtually constant over the entire temperature range analyzed, suggesting its high thermal stability. This behavior is attributed to the material having been previously sintered at 1000 °C, a process used to convert calcium-deficient HAp (CDHAp) into β-TCP. It is also likely to promote the complete removal of adsorbed water and potential volatile impurities. In contrast, HAp samples exhibited a modest weight loss between 100 and 400 °C, which is attributed to the release of water on the particle surfaces, possibly related to secondary bonding interactions.^40^ This observation is consistent with the fact that HAp is thermally less stable than β-TCP and may therefore contribute to a slight reduction in thermal stability and to an impact on rheological behavior.

Figure 4a presents the TGA profiles of the PCL-based composites, and Figure 4b shows the corresponding derivative curves (DTG). Figure 4a showed that the incorporation of ceramic fillers did not compromise the thermal stability of the PCL matrix. On the contrary, a slight increase in the onset degradation temperature (*T*_onset_) was observed with increasing filler content. The *T*_onset_ values, indicated in Table 3, were 384.7 °C for the PCL control, 385.2 °C for the PCL20TCP composite, 389.6 °C for PCL15TCP5HA, and 390.0 °C for PCL10TCP10HA. The modest increase in *T*_onset_ observed in the composites containing both HAp and β-TCP suggests a positive thermal stabilization effect promoted by the ceramic fillers. Unlike studies that report a reduction in thermal stability with the isolated incorporation of these additives,^33,41^ the results obtained here indicate that the adequate distribution of the particles may have acted as an effective thermal barrier, restricting the mobility of polymer chains and thereby delaying the irreversible degradation. Although similar stabilization effects have been described for isolated HAp or β-TCP fillers,^26,42,43^ studies evaluating the combined effect of these two ceramic phases in PCL matrices remain scarce, which reinforces the originality and relevance of the present findings. Still, these minor changes do not impair the 3D printing process.

**Figure 4:**
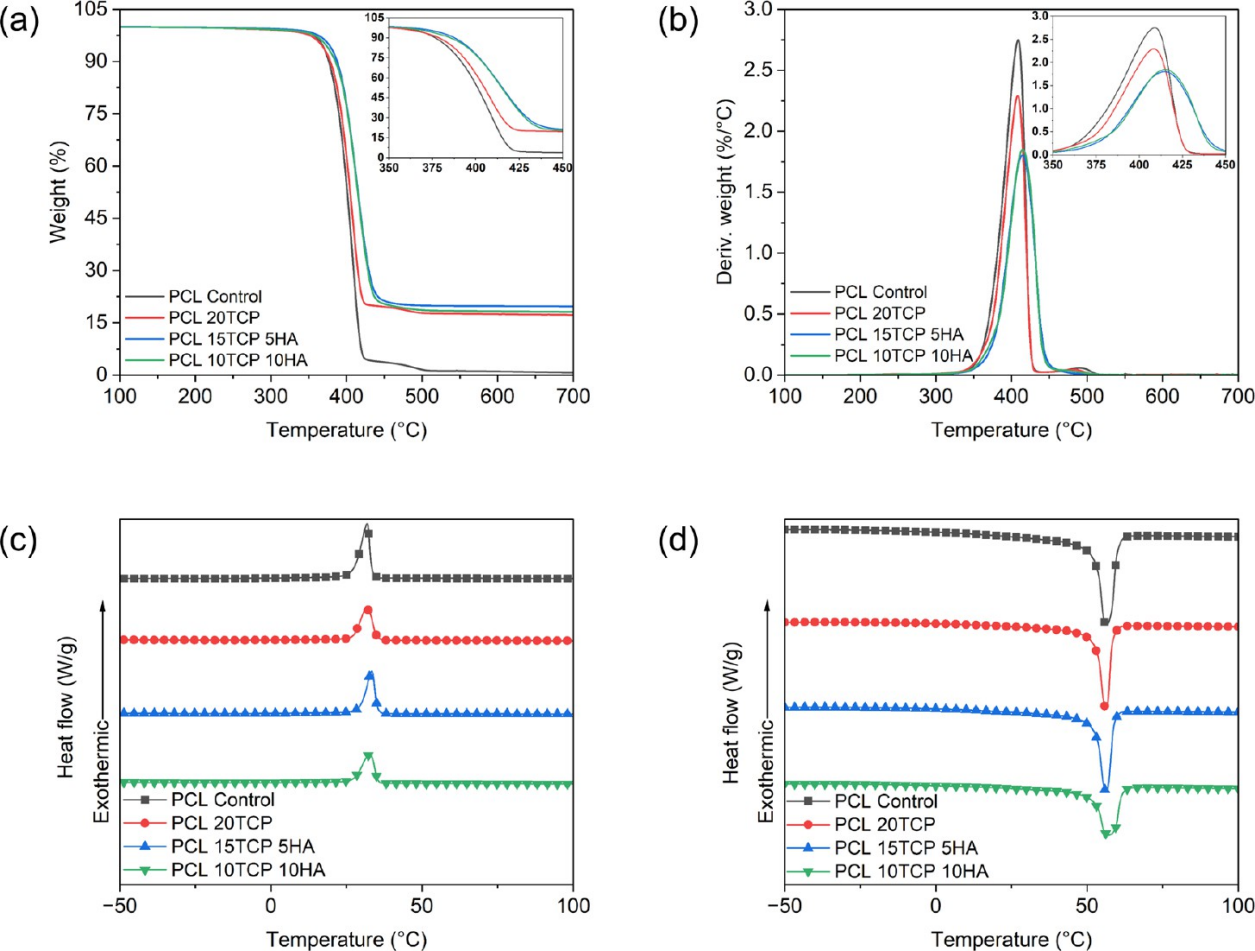
(a) Thermogravimetry and (b) derivative thermogravimetry curves of poly­(ε-caprolactone) and biocomposites; differential scanning calorimetry curves obtained in the (c) cooling and (d) second heating.

**Table 3:** Thermal Properties of Poly­(ε-caprolactone) and Composites with β-TCP and HAp^a^

sample	*T*_onset_ (°C)	*T*_max_ (°C)	ER (%)	TR (%)	*T*_m_ (°C)	*T*_c_ (°C)	ME (J.g^–1^)	DG (%)
PCL control	384.7	409.0	0.6	0.0	56.5	31.9	53.8	38.7
PCL20TCP	385.2	408.2	17.3	20.5	56.3	31.6	54.7	49.2
PCL15TCP5HA	389.6	414.5	19.6	19.9	56.4	33.3	58.6	52.7
PCL10TCP10HA	390.0	414.9	20.1	20.1	57.2	32.6	52.0	46.8

a DG: degree of crystallinity; ER: experimental residue; ME: melting enthalpy; TR: theoretical residue.

Between 350 and 450 °C, all samples exhibited significant weight loss attributed to the thermal decomposition of the polymer matrix, resulting from the cleavage of the main PCL chains. Furthermore, TGA analysis enabled the estimation of the ratio between the organic and inorganic phases in the composites. While PCL control was completely decomposed around 600 °C, leaving only 0.6% residue, the composites showed residual masses consistent with the inorganic calcium phosphate content: 17.3% for PCL20TCP, 19.6% for PCL15TCP5HA, and 18.1% for PCL10TCP10HA, close to theoretical values calculated using PCL and ceramic filler‘s residues at test end.

The DSC thermograms obtained during the cooling and second heating cycles are presented in Figure 4c,d, respectively, and the corresponding data are summarized in Table 3. The PCL sample exhibited a *T*_m_ of 56.5 °C and a Δ*H*_m_ of 53.8 J·g^–1^, resulting in a crystallinity degree of 38.7%, which aligns with the values reported in the literature for semicrystalline PCL.^44^ The other samples exhibited similar *T*_m_ values, ranging from 56.3 to 57.2 °C, indicating that the inclusion of ceramic fillers did not affect the melting temperature of the PCL matrix.

The crystallization temperature (*T*_c_) varied slightly among the formulations, with the PCL15TCP5HA sample exhibiting the highest *T*_c_ (33.3 °C), which may be attributed to a potential nucleating effect of the combined ceramic phases on polymer chain organization during cooling. Higher *T*_c_ values suggest that crystallization occurred more efficiently and at an earlier stage, indicating that the fillers had a positive influence on the structural ordering of the polymer. Regarding melting enthalpy (Δ*H*_m_), it is essential to note that the ΔH_m_ values for the composite samples were initially calculated based solely on the polymer fraction, excluding the ceramic fillers, which comprise 20 wt % of the total mass. Subsequently, the values were corrected by considering this percentage. Under this condition, all composites exhibited slightly higher Δ*H*_m_ compared to the control, indicating a minimal increase in the degree of molecular organization. The corrected values were 54.7 J·g^–1^ for PCL20TCP, 58.6 J·g^–1^ for PCL15TCP5HA, and 52.0 J·g^–1^ for PCL10TCP10HA. Among the modified samples, the PCL15TCP5HA formulation exhibited the highest crystallinity (52.7%), suggesting that an intermediate ratio of β-TCP to HAp favored polymer crystallization, possibly through a synergistic nucleation effect between the two ceramic fillers. It is believed that the combination of these reinforcements promoted a more effective distribution of heterogeneous nucleation sites, thereby facilitating the organization of polymer chains during cooling. In contrast, although the PCL10TCP10HA sample remained more crystalline than the PCL control, it exhibited slightly lower crystallinity than the other composite formulations (46.8%). This behavior may be related to the distinct surface characteristics of HAp, which, due to its high specific surface area and reactivity, may have acted as an efficient nucleating agent, promoting the onset of crystallization at higher temperatures. However, its higher proportion relative to β-TCP may have compromised the efficiency of the crystalline network formation and resulted in lower overall crystallization.

These findings indicate that the presence and ratio of ceramic phases influenced the crystallinity of PCL in a composition-dependent manner, without significantly affecting the thermal transition temperatures. Similar trends have been reported, supporting the idea that interactions between the fillers and the polymer matrix can modulate the semicrystalline structure of the system.^45,46^

### 3.4 Atomic Force Microscopy

To further evaluate the effects of surface modification on scaffold topography, AFM was used to examine representative samples before and after surface treatment. As shown in Figure 5, the untreated scaffold exhibited a relatively smooth and uniform surface, consistent with the characteristics of the as-fabricated polymeric matrix. In contrast, the sample treated with 1 mol·L^–1^ NaOH for 8 h exhibited increased nanoscale surface roughness, characterized by etched features and irregular elevations. These modifications indicate polymer chain hydrolysis and matrix degradation, thereby facilitating the exposure of embedded bioactive ceramic particles. The observed increase in surface complexity is expected to enhance protein adsorption and cell attachment, thereby improving the scaffold’s bioactivity and biological performance. According to the review by Backes et al.,^47^ alkaline surface treatments, particularly those using NaOH, are effective strategies for modifying the nanoscale topography of PCL-based scaffolds. The authors highlighted that NaOH induced polymer chain hydrolysis and controlled matrix degradation, thereby increasing surface roughness and exposing embedded bioactive fillers.

**Figure 5:**
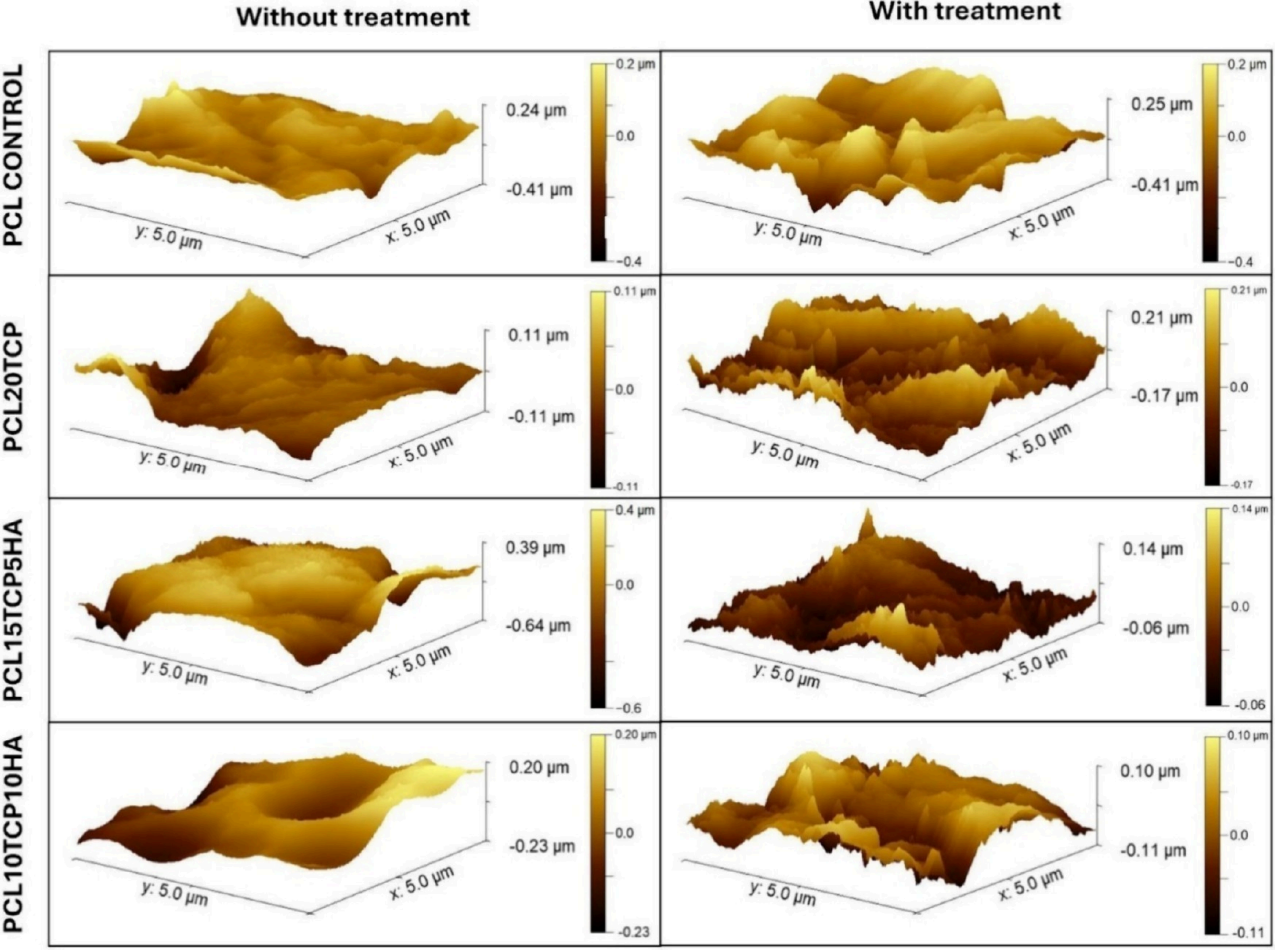
Atomic force microscopy (AFM) topographic images comparing scaffold surfaces before and after treatment.

These surfaces topographical changes observed via AFM are in agreement with previous reports from Lam et al.*,* ^23^ who showed that alkaline treatment of PCL−β-TCP scaffolds with NaOH resulted in microscale surface degradation and formation of pores due to the preferential removal of the amorphous polymer regions and exposure of β-TCP particles. Similarly, Wang et al.^24^ demonstrated that NaOH treatment on uniaxially stretched PCL films induced increased surface roughness, as confirmed by AFM, which in turn facilitated improved cell alignment and anchorage. These findings support the conclusion that NaOH surface etching effectively enhances surface roughness across different PCL-based systems, providing a favorable topography for cellular interaction and tissue integration.

### 3.5 Protein Adhesion

The results indicate a significant increase in protein (BSA) adsorption in all groups subjected to NaOH treatment, compared to their untreated counterparts. Notably, the PCL15TCP5HA_T, and PCL10TCP10HA_T groups showed the highest BSA adsorption, suggesting that surface modification effectively enhanced protein affinity. These morphological modifications also enhanced protein adsorption, as can be observed in Figure 6. Chemical etching with NaOH promoted the hydrolysis of PCL’s ester bonds, resulting in the formation of charged surfaces of the materials. At physiological pH, these charged groups could promote the electrostatic attraction of proteins. This initial interaction contributed to the proper orientation of the adsorbed proteins, creating an environment more conducive to cell adhesion and proliferation.^48,49^ Also, a more irregular surface, is known to aid protein attachment and ultimately facilitating cell attachment, contributing to improved scaffold bioactivity and biological performance in bone tissue engineering applications.^50,51^

**Figure 6:**
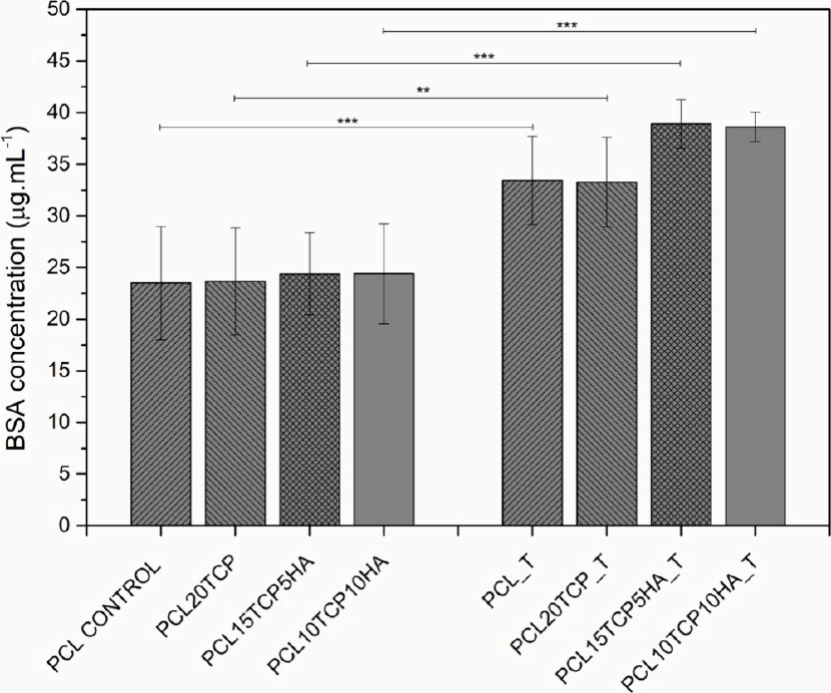
Protein adhesion assay for scaffold before and after surface treatment (T) (*n* = 5; *: *p* < 0.05; **: *p* < 0.01; ***: *p* < 0.005).

### 3.6 Microstructural and Porosity Characterization

In composite scaffolds, ceramic particles are often encapsulated within the polymeric matrix during processing, limiting surface exposure and thereby reducing biological performance, particularly in bioceramics. To overcome this drawback, a surface treatment using 1 M sodium hydroxide (NaOH) for 8 h was applied. This simple, low-cost, efficient, and nontoxic method partially etches the polymer surface, thereby exposing the ceramic particles and enhancing their interfacial availability. Such modification is intended to improve cell-material interactions and promote better tissue integration. Figure 7 shows SEM images of the scaffolds, taken under three different experimental conditions, enabling a comparative analysis of surface morphology and porosity after treatment.

**Figure 7:**
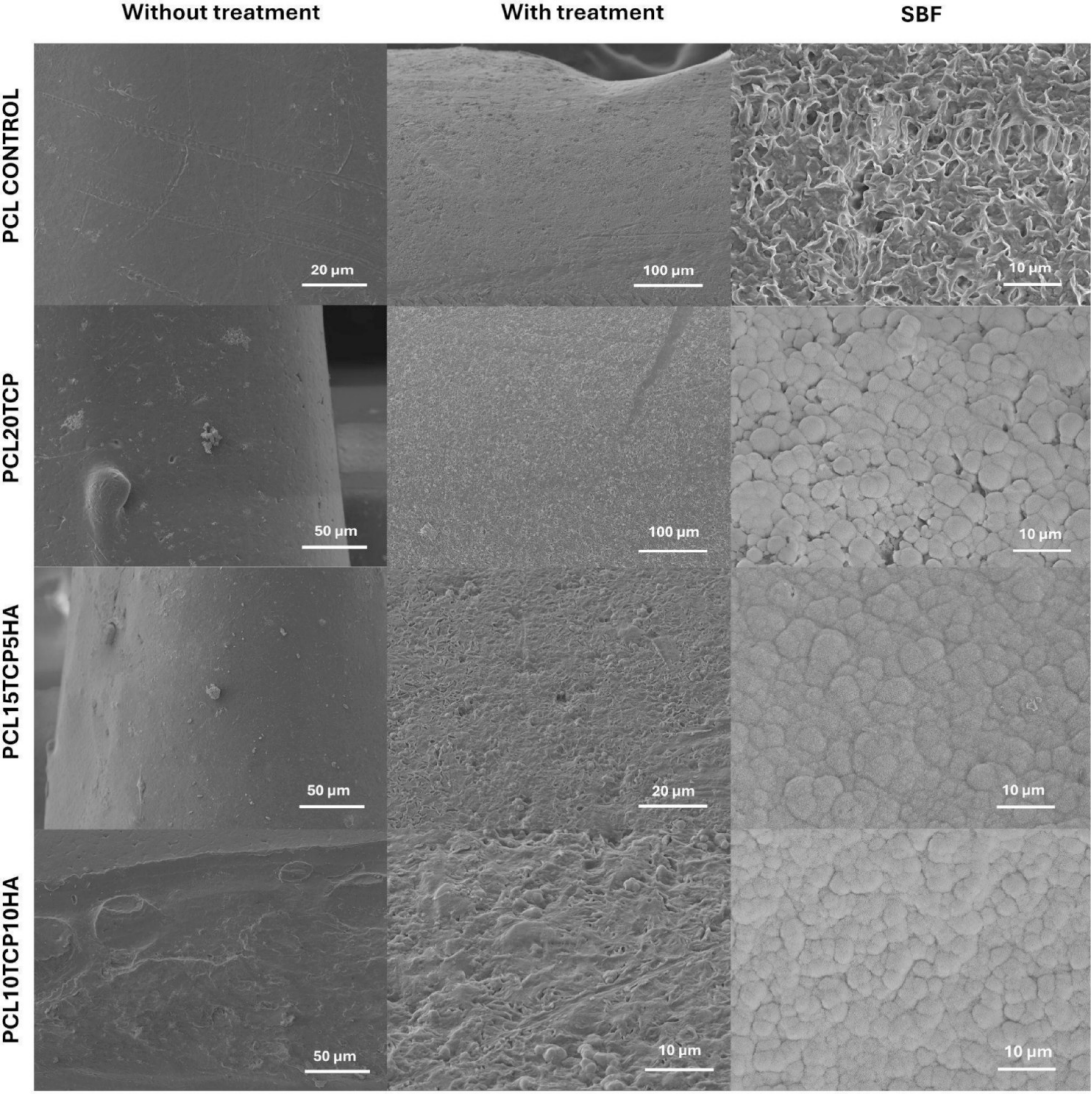
SEM micrographs of the scaffolds for each composition under three different experimental conditions. Left column: as-fabricated scaffolds. Middle column: scaffolds after surface treatment with 1 mol·L^–1^ NaOH for 8 h. Right column: NaOH-treated scaffolds after immersion in simulated body fluid (SBF) for 7 days.

The left column corresponds to the as-fabricated scaffolds, which exhibited a relatively smooth surface morphology characteristic of polymer-based matrices. In these samples, the embedded ceramic fillers were not visibly exposed, indicating that they were fully encapsulated within the polymeric phase. According to Joseph et al.,^52^ the as-printed PCL/HAp scaffolds typically exhibited a smooth surface morphology and an enclosed pore architecture, with bioactive fillers, such as HAp, remaining embedded within the polymeric phase. This observation aligns with the initial characterization of the untreated samples, in which ceramic particles were not visibly exposed on the scaffold surface.

The middle column presented the scaffolds after surface treatment with 1 mol·L^–1^ NaOH for 8 h. A more pronounced surface degradation was observed, resulting in a roughened and irregular topography. This alkaline hydrolysis process effectively removed superficial polymer layers, revealing previously encapsulated ceramic particles and creating microcavities within the material. Mass variation analysis of the scaffolds after NaOH treatment showed no significant difference, suggesting that the alkaline treatment did not cause macroscopic degradation of the material (Figure S2). Similarly, monitoring the pH of PBS solutions containing the scaffolds over 7 days showed no significant changes, indicating the system’s chemical stability during this period (Figure S3). Such morphological changes are advantageous for subsequent bioactivity, as they increase surface area and expose functional groups that can interact with the physiological environment. In a previous study by Backes et al.,^12^ the surface treatment of PCL/HAp scaffolds with a NaOH solution significantly altered the surface morphology by selectively etching the polymer matrix. This alkaline hydrolysis not only increased surface roughness but also exposed previously embedded TCP and HAp particles, generating microcavities that promoted enhanced cell adhesion and proliferation. These morphological modifications were demonstrated to be beneficial for bone tissue engineering applications, as they increased the surface area and enhanced the biointerfacial properties of the scaffolds.

In the right column, the scaffolds were immersed in SBF. In the compositions with HAp and TCP, the formation of a cauliflower structure composed of calcium phosphate deposits was clearly evident, especially in regions previously modified by the NaOH treatment. This indicates a positive bioactive response, suggesting that the surface activation achieved by alkaline treatment promoted nucleation and growth of apatite-like phases. The presence of these mineral deposits is consistent with in vitro biomineralization, which is a desirable feature for scaffolds intended for bone tissue regeneration. This suggested that the alkaline treatment enhances nucleation sites for mineral deposition. Such biomineralization behavior is indicative of a favorable in vitro response, reinforcing the suitability of these scaffolds for bone tissue engineering applications.^53^

To complement the morphological assessment, Figure 8 shows the EDS mapping of scaffold cross sections, highlighting the spatial distribution of HAp and TCP. The analysis confirmed a relatively homogeneous dispersion of inorganic particles within the polymeric matrix, suggesting that the fabrication method effectively ensured filler incorporation without significant agglomeration. These results demonstrate the effectiveness of the surface treatment strategy in enhancing scaffold bioactivity and confirm the potential of the developed composites for applications in periodontal or bone tissue engineering. According to a previous study by our team,^54^ EDS mapping of PLA-based scaffolds containing β-TCP and cerium oxide nanoparticles revealed a uniform distribution of the inorganic fillers throughout the polymeric matrix. The absence of significant particle agglomeration confirmed the effectiveness of the melt extrusion and 3D printing process in producing homogeneous composite filaments. These results support the notion that a well-dispersed phase enhances the interaction between bioactive particles and the surrounding environment, thereby improving scaffold bioactivity and reinforcing their suitability for applications in periodontal tissue engineering.

**Figure 8:**
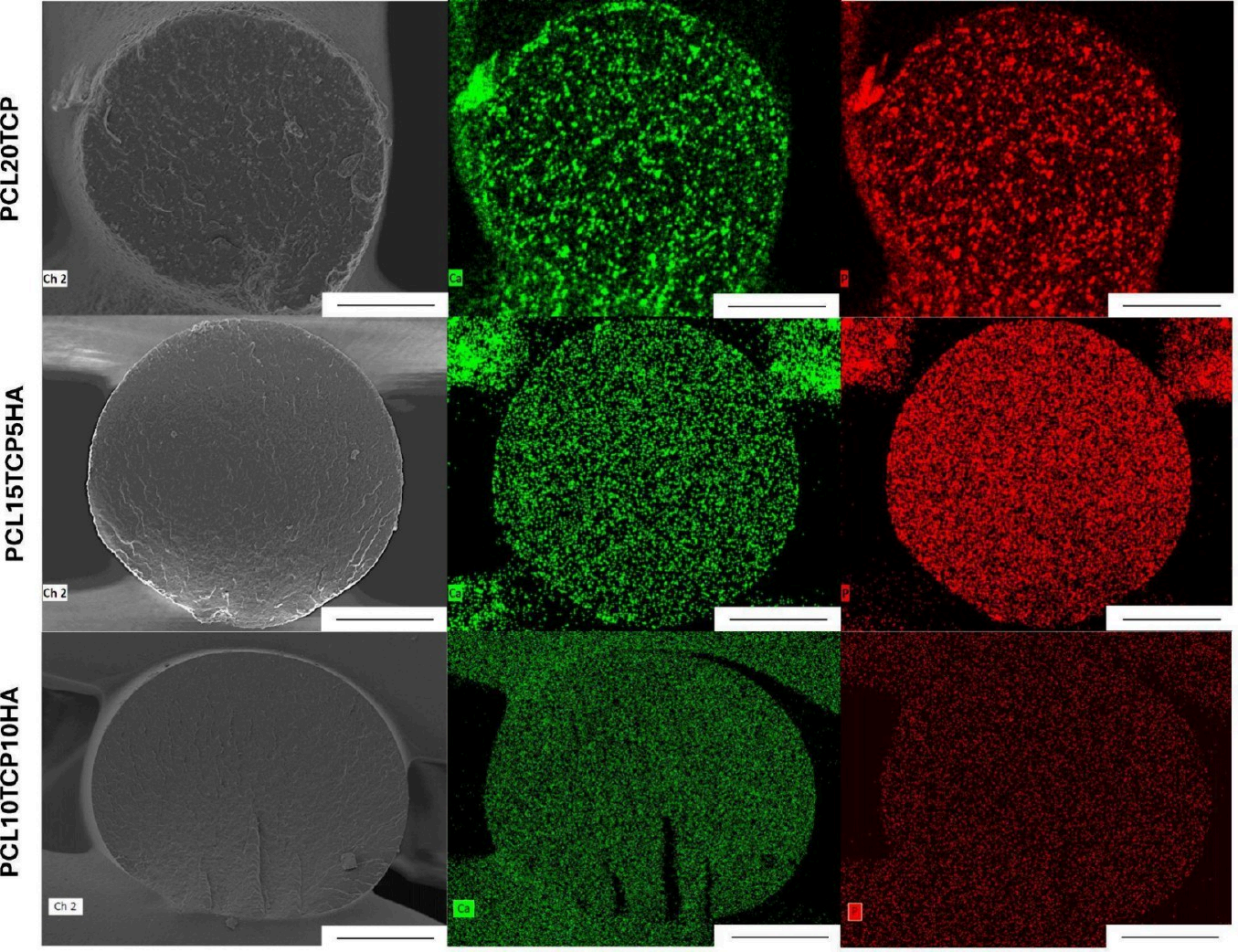
Energy dispersive spectroscopy mapping of scaffold cross sections showing the distribution of ceramic fillers within the polymeric matrix (green: calcium; red: phosphorus; scale 100 μm).

Table 4 presents quantitative measurements of pore size, strut width, and strut height for the scaffolds, obtained from detailed image analysis of high-resolution SEM micrographs. The as-fabricated PCL scaffolds exhibited a mean pore size of 385 ± 42 μm, with strut widths and heights of 351 ± 43 and 357 ± 47 μm, respectively. These dimensional values are in close agreement with the original computer-aided design (CAD) parameters, thus confirming the high geometric fidelity and reproducibility of the additive manufacturing process. The slight discrepancies observed may be ascribed to factors inherent to the FFF technique, such as minor instabilities in melt flow rate, thermal gradients at the nozzle–substrate interface, or slight inconsistencies in extrusion pressure and nozzle movement synchronization. Moreover, the rapid cooling of the extruded polymer and its viscoelastic behavior during deposition may induce subtle distortions in strut geometry. Despite these variations, the results demonstrated the precision of the printing setup and validated the suitability of the processing conditions for fabricating scaffolds with controlled architecture, which is crucial for ensuring mechanical reliability and biological performance in tissue engineering applications.^52^

**Table 4:** Morphological Parameters of 3D-Printed Scaffolds Before and After Surface Treatment^a^

material	pore size (μm)	strut width (μm)	strut height (μm)
PCL control	385 ± 42	351 ± 43	357 ± 47
PCL_T	384 ± 41	316 ± 21	309 ± 29
PCL20TCP	353 ± 16	314 ± 29	285 ± 8
PCL20TCP_T	372 ± 39	320 ± 20	309 ± 32
PCL15TCP5HA	376 ± 24	310 ± 17	315 ± 21
PCL15TCP5HA_T	381 ± 22	329 ± 31	289 ± 17
PCL10TCP10HA	361 ± 52	323 ± 17	293 ± 16
PCL10TCP10HA_T	345 ± 45	344 ± 32	304 ± 38

a Values correspond to mean ± SD for pore size, strut width, and strut height.

After surface treatment, a slight reduction in strut dimensions was observed, likely due to mild alkaline hydrolysis of the polymer matrix, while the pore size remained essentially unchanged. This indicates that the treatment selectively modified the strut surfaces without altering the overall macroporosity. The incorporation of ceramic fillers affected the structural definition of the printed scaffolds, with higher β-TCP content resulting in more pronounced dimensional changes and reduced print fidelity. These effects align with rheological behavior, where increased viscoelastic stiffness and reduced flowability can hinder proper layer deposition. Formulations containing HAp exhibited comparatively lower viscosities, which favored more accurate printing. Surface treatment further induced architectural adjustments, such as partial recovery of strut dimensions or densification of the printed structure, depending on the filler composition. Overall, both filler content and postprocessing emerge as key parameters for tailoring scaffold architecture, with direct implications for mechanical integrity and biological performance.

### 3.7 Mechanical Characterization

Figure 9 shows the compressive stress–strain curves obtained for the fabricated scaffolds before (a, b) and after (c, d) surface treatment. In general, all samples exhibited a typical mechanical behavior expected for porous polymeric structures, characterized by an initial linear elastic region followed by a plateau associated with the progressive collapse of the porous architecture.

**Figure 9:**
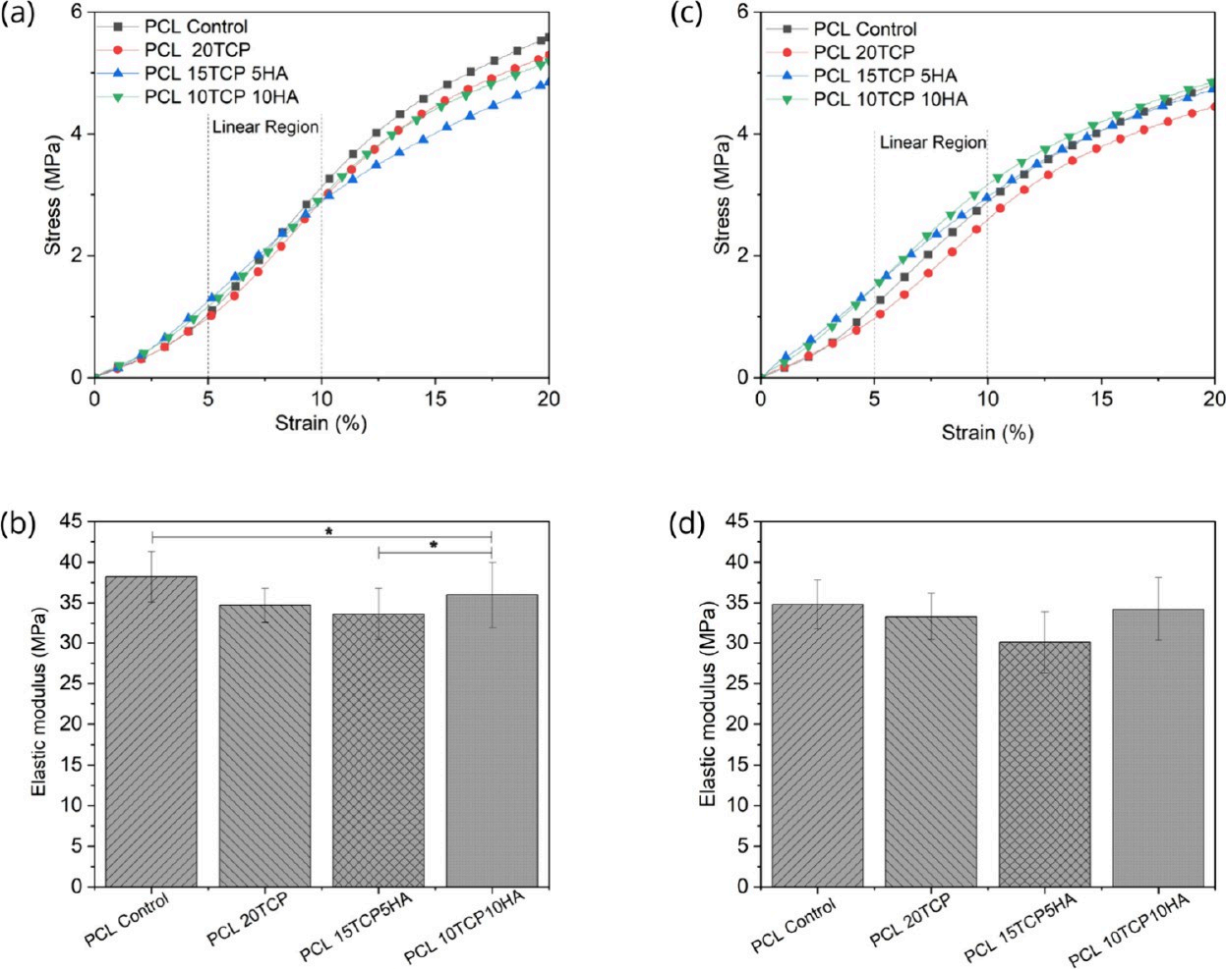
Compressive stress–strain curves of the scaffolds: (a, b) untreated samples; (c, d) scaffolds after alkaline surface treatment (*n* = 5; *: *p* < 0.05; **: *p* < 0.01; ***: *p* < 0.005).

In the untreated samples (Figure 9a,b), the mechanical behavior varied significantly with composition. Pure PCL scaffolds exhibited high deformability and one of the highest elastic moduli, indicating a favorable combination of flexibility and stiffness. The incorporation of ceramic fillers (TCP and HAp) altered the mechanical response, generally reducing the elastic modulus, particularly in the PCL15TCP5HA group, which showed the lowest among all compositions. This suggests that, rather than reinforcing the structure, the combined presence of both calcium phosphate phases at this ratio may have interfered with effective load transfer within the polymer matrix. Notably, the PCL10TCP10HA group demonstrated mechanical properties comparable to those of pure PCL, suggesting a potentially more balanced distribution of reinforcement. These findings reinforce the notion that scaffold stiffness must be carefully tailored to avoid mechanical mismatches and suboptimal cellular responses. As highlighted by Guimarães et al.,^55^ the stiffness of engineered constructs not only affects their structural integrity but also plays a critical role in cell mechanotransduction, influencing key processes such as adhesion, migration, and differentiation. Therefore, the observed decrease in stiffness in some composite formulations may have implications beyond mechanical performance, potentially compromising the scaffold’s ability to support effective tissue regeneration. Niu et al.^56^ demonstrated that the mechanical performance of 3D-printed PCL/β-TCP scaffolds was highly dependent on the proportion of the ceramic phase, with certain ratios enhancing compressive strength while others compromising it due to uneven phase distribution or poor interfacial bonding. In particular, they highlighted that optimized formulations could promote both mechanical reinforcement and favorable biological responses. In light of this, the inferior stiffness observed in the PCL15TCP5HA group may result from an imbalance between the two ceramic fillers. In contrast, the PCL10TCP10HA composition appears to more closely resemble the synergistic effect described by Guimarães et al.^55^ supporting both structural and functional outcomes.

After the alkaline surface treatment (Figure 9c,d), a general trend of reduced mechanical performance was observed, particularly in compressive strength and strain at break. This reduction can be attributed to the superficial hydrolysis of PCL ester bonds, leading to a rougher, more brittle surface layer. Despite this, the treated PCL15TCP5HA_T samples maintained relatively higher mechanical integrity, indicating that the ceramic phase also contributes to counteracting the degradation-induced weakening. These findings emphasize that while surface treatment enhances bioactivity, it may compromise the mechanical robustness of the scaffolds. Therefore, a careful balance must be struck between biofunctionalization strategies and the preservation of structural integrity, particularly in applications that require mechanical support during the early stages of bone regeneration. Similar concerns have been highlighted by Hedayati et al.,^57^ who demonstrated that modifications aimed at accelerating biodegradation, such as incorporating biodegradable fiber yarns into PCL scaffolds, can significantly reduce mechanical strength over time. Their results underscore the importance of controlling degradation mechanisms to ensure the scaffold maintains sufficient mechanical performance throughout the critical phases of tissue healing.

In addition, it is acknowledged that mechanical evaluation under simulated physiological conditions could provide complementary insights into the behavior of the composites. However, the tests conducted under dry conditions already provided a clear assessment of the influence of ceramic fillers and NaOH surface treatment on the structural integrity of PCL-based composites. Nevertheless, future studies are planned to include dynamic mechanical analysis (DMA) under PBS immersion to investigate the effect of hydration on the mechanical response at different degradation time points, as well as to correlate these findings with mass-loss analyses and more comprehensive in vitro studies involving various primary cell types.

### 3.8 Hydrophilicity and Biological Assays

To evaluate the surface wettability of the scaffolds before and after alkaline treatment, contact angle measurements were performed for all compositions (Figure 10). The contact angle measurements demonstrated a consistent reduction in water contact angle for all samples treated with 1 M NaOH, indicating enhanced surface hydrophilicity. This effect is attributed to the partial hydrolysis of the ester bonds in the PCL chains, which leads to the formation of hydrophilic functional groups, such as hydroxyl and carboxyl groups, on the surface of the scaffold. Moreover, Joseph et al.^52^ emphasized that enhancing scaffold surface energy via chemical modification is essential to promote biomolecular interactions, especially in hydrophobic polymers such as PCL. In the present study, the reduction of contact angle values in all treated groups corroborates these findings and confirms the effectiveness of the alkaline treatment in improving scaffold wettability, regardless of the ceramic filler composition. This surface modification is particularly relevant for early stage cell-scaffold interactions, protein binding, and subsequent tissue regeneration processes.

**Figure 10:**
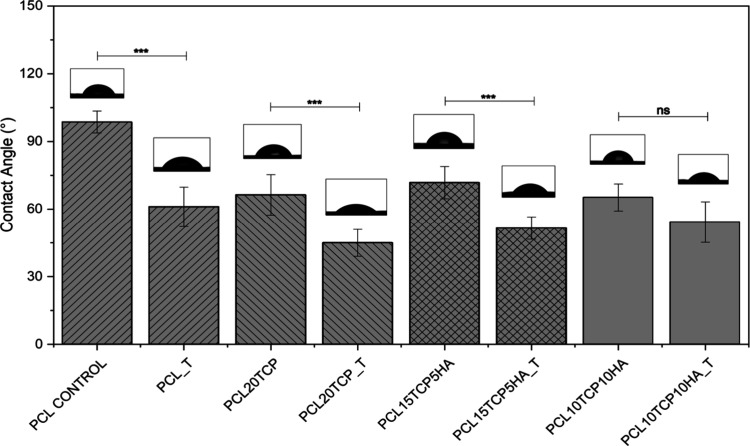
Contact angle measurements of poly­(ε-caprolactone)-based before and after alkaline treatment (T). The incorporation of ceramic fillers (β-TCP and HAp) and the surface modification significantly influenced the wettability (*n* = 5; *: *p* < 0.05; **: *p* < 0.01; ***: *p* < 0.005).

To investigate the biological performance of the scaffolds, in vitro assays were conducted using murine preosteoblastic MC3T3-E1 cells. Cell viability and metabolic activity were quantified using the resazurin-based assay after 7 days of culture, while cell adhesion and morphology on the scaffold surfaces were qualitatively evaluated after 24 h by SEM. The results of these biological evaluations are shown in Figure 11.

**Figure 11:**
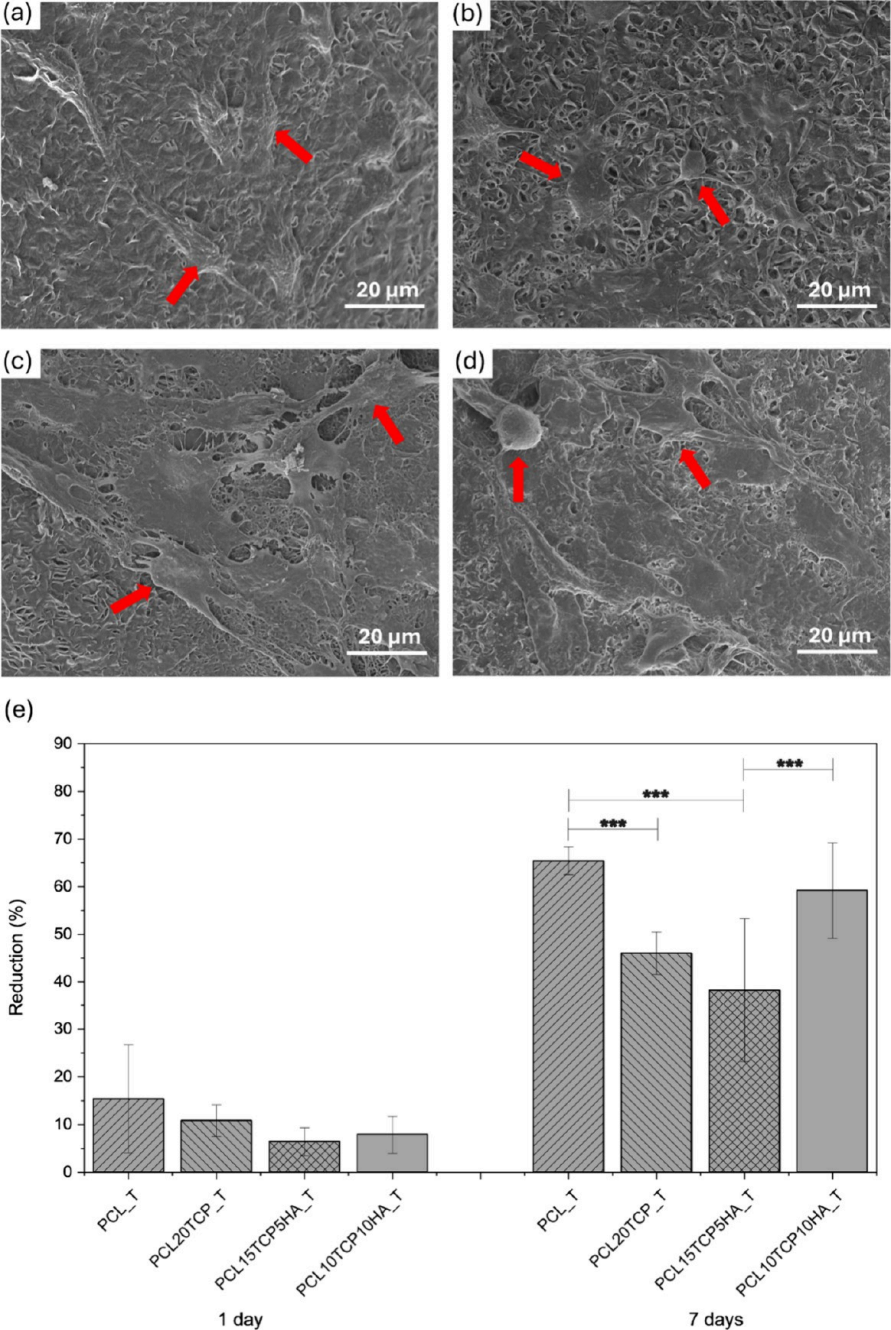
Biological evaluation of poly­(ε-caprolactone)-based scaffolds. Scanning electron microscopy images (a–d) show the morphology and attachment of cells on (a) PCL control, (b) PCL20TCP, (c) PCL15TCP5HA, and (d) PCL10TCP10HA after 24 h of culture. (e) Cell viability assay for analysis on days 1 and 7 of incubation of the scaffolds, indicated as percentage of metabolic activity reduction for NaOH-treated scaffolds (*n* = 5; *: *p* < 0.05; **: *p* < 0.01; ***: *p* < 0.005).

The biological performance of the NaOH-treated scaffolds was evaluated using MC3T3-E1 preosteoblastic cells through a combination of qualitative (SEM) and quantitative (resazurin-based) assays. The bar graphs in Figure 10e present the percentage of resazurin reduction after 1 and 7 days of cell culture, reflecting changes in metabolic activity and, indirectly, cell proliferation over time. In parallel, SEM micrographs show cell attachment and morphology after 24 h of seeding on different scaffold compositions (Figure 11a–d). After 1 day, all compositions exhibited relatively low metabolic activity, as expected for the early stage of cell adhesion and proliferation. However, after 7 days, a significant increase in resazurin reduction was observed across all groups, indicating a time-dependent rise in cell metabolic activity and confirming that the scaffolds support osteoblastic proliferation over time. The most prominent increase was observed in the PCL control group treated with NaOH, indicating that, despite the absence of ceramic reinforcement, the alkaline treatment sufficiently improved the surface conditions (e.g., hydrophilicity and roughness) to promote sustained cellular growth.

Nevertheless, scaffolds containing ceramic fillers, particularly PCL 15TCP5HA_T and PCL 10TCP10HA_T, also demonstrated robust increases in metabolic activity over the 7-day period. This is consistent with the literature, which reports that β-TCP and HAp not only contribute to osteoconductivity but also provide a more favorable microenvironment for cell adhesion, mineralization, and differentiation due to their ionic release and buffering capacity. The SEM images further corroborated the above-mentioned results. After 24 h, cells on all scaffold types displayed well-spread morphology, with filopodia extending into the porous microstructure. This suggests that the NaOH-treated surfaces facilitated proper initial cell attachment, a prerequisite for subsequent proliferation and differentiation. In particular, the PCL15TCP5HA_T and PCL10TCP10HA_T scaffolds exhibited more extensive cellular coverage and interaction with the scaffold surface, suggesting a synergistic effect between surface treatment and ceramic phase composition.

Recent developments in regenerative medicine have expanded the understanding of how biomaterials can guide cell behavior and enhance tissue repair. Han et al.^58^ provided a comprehensive overview of stem cell–based strategies for periodontal regeneration, emphasizing the synergistic role of stem cells and bioengineered scaffolds in recreating functional tissue architecture. Their findings highlighted that scaffold composition, surface properties, and degradation kinetics are essential parameters to control osteogenic differentiation and periodontal attachment formation. Recent research has demonstrated that not only the composition but also the crystalline organization of polymeric scaffolds can play a decisive role in their performance for bone and periodontal regeneration. Huang et al.^59^ provided compelling evidence that controlled crystal growth within 3D-printed PCL scaffolds significantly enhanced their mechanical strength, surface energy, and biological response. By manipulating crystallization kinetics during fabrication, the authors achieved scaffolds with hierarchical structures that promoted improved cell adhesion and proliferation without compromising elasticity. These findings underscored how tuning crystallinity and molecular alignment in biodegradable polymers could optimize both the material’s physicochemical and biological functions.

Overall, the results indicate that alkaline-treated scaffolds, regardless of their ceramic content, can support the proliferation of preosteoblastic cells over time. However, the combination of surface hydrophilicity, microscale roughness, and bioactive ceramic phases appears to enhance not only initial adhesion but also long-term cellular response, reinforcing the importance of compositional and surface engineering in scaffold design for periodontal tissue engineering. Further analysis using mesenchymal stem cells will be performed, aiming to assess the differentiation potential of combined HAp and TCP ceramic fillers on NaOH-treated PCL biocomposite scaffolds.

## 4 Conclusions

This study demonstrates that combining ceramic fillers—β-tricalcium phosphate (β-TCP) and hydroxyapatite (HAp)—with alkaline surface treatment via NaOH is an effective strategy for enhancing the bioactivity of 3D-printed poly­(ε-caprolactone) (PCL) scaffolds intended for periodontal tissue regeneration. The incorporation of ceramic fillers directly influenced not only the bioactivity but also the rheological and printing behavior of the composite, with distinct effects of each filler on melt viscosity and viscoelastic properties. These rheological parameters critically governed the fidelity of the printed scaffolds, determining strut resolution, pore geometry, and dimensional stability. Microscopic and topographical analyses, including atomic force microscopy (AFM), revealed that alkaline treatment selectively hydrolyzed the polymer surface, increasing roughness and facilitating the partial exposure of embedded ceramic particles. These modifications occurred without altering the scaffold’s interconnected macroporosity. The enhanced roughness and heterogeneous microtopography created a favorable environment for protein adsorption, as confirmed by BCA assay results. Notably, scaffolds subjected to NaOH treatment exhibited significantly higher protein binding capacity, suggesting increased surface energy and functional group availability that promote protein–material interactions. Filler characterization confirmed that both β-TCP and HAp particles presented submicron dimensions, which favor surface exposure and may contribute to enhanced local bioactivity. While filler dispersion within the matrix was not directly evaluated, the observed biological responses suggest effective particle–cell interaction at the scaffold surface. These particles contributed not only to osteoconductivity but also played a role in modulating mechanical behavior. Despite the chemical surface modifications and ceramic integration, compressive testing indicated that the mechanical integrity—specifically compressive strength and stiffness—remained uncompromised, maintaining suitability for periodontal applications. In vitro biological assays using MC3T3-E1 preosteoblastic cells confirmed that all formulations were cytocompatible, supporting cell adhesion, spreading, and proliferation. The improved biological response is attributed to the synergistic effects of increased surface roughness, ceramic bioactivity, and protein adsorption capacity. Collectively, these results highlight the potential of combining material composition control, 3D-printing parameters, and surface post-treatment as a multifactorial design strategy for bioactive scaffolds with balanced mechanical and biological performance. Future investigations will extend this approach using different cell lines and in vivo models to further validate its translational relevance in regenerative dentistry and bone tissue engineering.

## Supplementary Material



## Notes

Thermogravimetry curves of HAp and β-TCP; mass loss (%) of poly­(ε-caprolactone)-based composites after degradation testing; and pH variation of poly­(ε-caprolactone)-based composites after 7 days, without and with treatment (PDF)
