# Supplementary material for: Synergistic Effects of Ceramic Fillers and NaOH Treatment on Bioactivity of 3D-Printed Poly(ε-caprolactone) Scaffolds for Periodontal Tissue Regeneration
Source: ACS Omega. 2026 Jan 19;11(4):5508–25. doi: 10.1021/acsomega.5c09061 (PMC12878396; doi:10.1021/acsomega.5c09061)
Supplement: Supplementary file 1 [file ao5c09061_si_001.pdf]

Synergistic effects of ceramic fillers and NaOH treatment on bioactivity of 3D-printed Poly (ε-caprolactone) scaffolds for periodontal tissue regeneration

João de Freitas Gomes Neto[1], Bruno Pinto Moura[2] Tainara de Paula de Lima Lima[1], Felippe Migliato Marega[1], Leonardo Alves Pinto[1], Pedro Lopes Granja[3], Luiz Antonio Pessan[1-2], Eduardo Henrique Backes[1-3]

[1] Federal University of São Carlos, Graduate Program in Materials Science and Engineering, São Carlos, SP, Brazil.

[2] Department of Materials Engineering, 13565-905, Brazil, UFSCar, São Paulo, Brazil.

[3] Biofabrication Group, i3S - Instituto de Investigação e Inovação em Saúde (i3S), Universidade do Porto, Porto, Portugal.

1. Supplementary Results

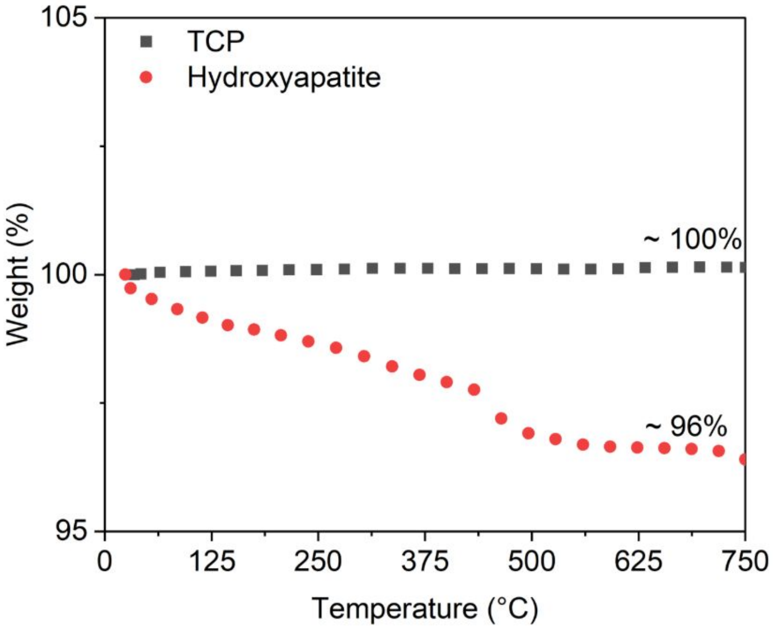

Figure S1 - Thermogravimetry curves of HAp and β-TCP.

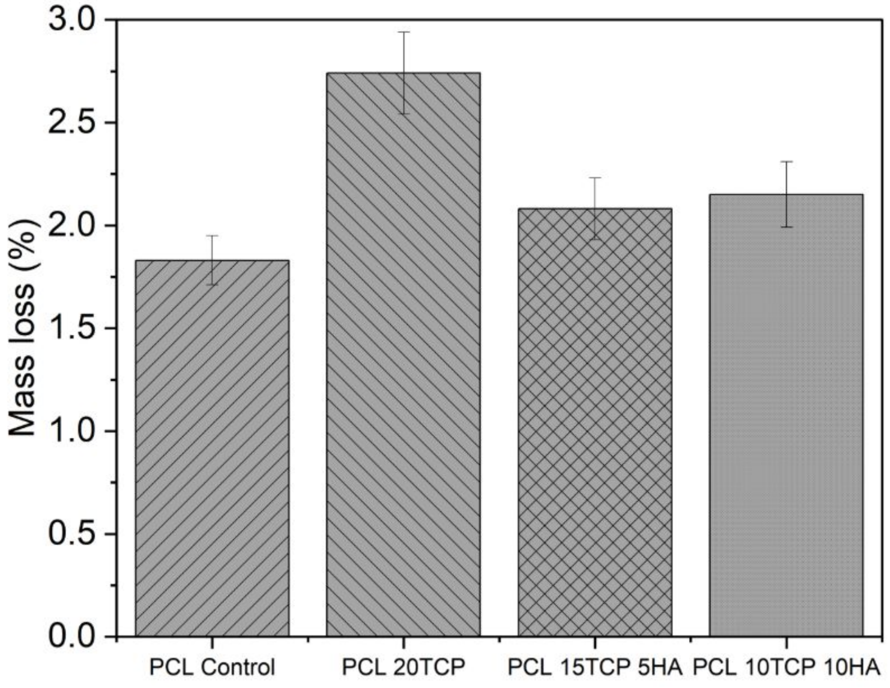

Figure S2 - Mass loss (%) of Poly (ε-caprolactone)-based composites after degradation testing.

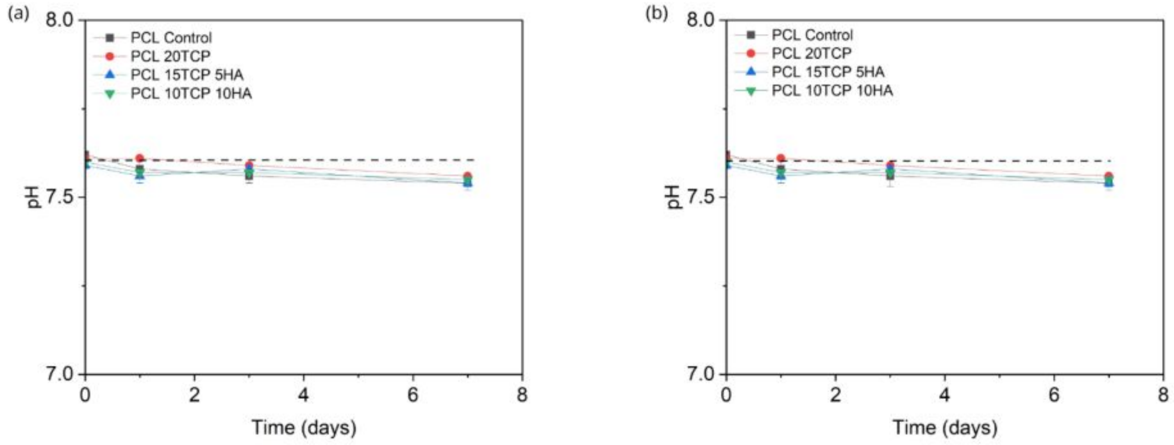

Figure S3 - pH variation of Poly (ε-caprolactone)-based composites after 7 days: (a) without treatment; and (b) treated.
